# Elucidation of the relationship between sensory processing and white matter using diffusion tensor imaging tractography in young adults

**DOI:** 10.1038/s41598-021-91569-6

**Published:** 2021-06-08

**Authors:** Daichi Shiotsu, Minyoung Jung, Kaie Habata, Taku Kamiya, Ichiro M. Omori, Hidehiko Okazawa, Hirotaka Kosaka

**Affiliations:** 1 Department of Neuropsychiatry, University of Fukui, Eiheiji, Japan; 2 Cognitive Science Group, Korea Brain Research Institute, Daegu, South Korea; 3 Biomedical Imaging Research Center, University of Fukui, Eiheiji, Japan; 4 Research Center for Child Mental Development, University of Fukui, Eiheiji, Japan; 5 Division of Developmental Higher Brain Functions, Department of Child Development, United Graduate School of Child Development, University of Fukui, Eiheiji, Japan

**Keywords:** Neuroscience, Medical research

## Abstract

Sensory processing and behaviors are altered during the development of connectivity between the sensory cortices and multiple brain regions in an experience-dependent manner. To reveal the relationship between sensory processing and brain white matter, we investigated the association between the Adolescent/Adult Sensory Profile (AASP) and neural connectivity in the white matter tracts of 84 healthy young adults using diffusion tensor imaging (DTI). We observed a positive relationship between AASP scores (i.e., sensory sensitivity, sensation avoiding, activity level)/subscores (i.e., sensory sensitivity–activity level, sensation avoiding–touch) and DTI parameters in the cingulum–cingulate gyrus bundle (CCG) and between AASP subscores (i.e., sensory sensitivity–auditory) and a diffusion parameter in the uncinate fasciculus (UNC). The diffusion parameters that correlated with AASP scores/subscores and AASP quadrant scores (i.e., sensory avoiding and sensitivity) were axonal diffusivity (AD) and mean diffusivity (MD) in the CCG and MD in the UNC. Moreover, the increased sensory avoiding and sensitivity scores represent the sensitization of sensory processing, and the level of diffusivity parameters indicates white matter microstructure variability, such as axons and myelin from diffusivity of water molecules. Thus, the present study suggests that the CCG and UNC are critical white matter microstructures for determining the level of sensory processing in young adults.

## Introduction

Sensory processing is a physiological response to sensory events evoked by stimuli encountered in the environment^1^. For example, sensory processing provides social information by integrating visual stimuli such as a gaze, facial expressions, and biological motion^2^, and it affects daily living skills and consequently quality of life^3,4^. A previous study indicated that differences in sensory processing, such as abnormal sensory sensitivity and low–sensory registration, may disturb motor development and are associated with the learning of daily living skills, such as those required for bathing and using the toilet^5^.

Dunn proposed a theory of sensory processing explaining individual variations in behavioral response^6^. In Dunn’s theory, two principal axes (i.e., the neurological threshold and behavioral response) comprise the foundation of responses to sensory stimuli. The neurological threshold denotes that stimuli are slowly or quickly noticed. Additionally, a passive behavioral response allows sensory experiences to occur, inciting a reaction, followed by an active behavioral response allowing behavior engagement to control sensory input. According to these two principal axes, the response to sensory stimuli are divided into four quadrants: low registration, sensation seeking, sensory sensitivity, and sensation avoiding^7^. Following Dunn’s theory, the Sensory Profile Questionnaire was developed to quantify individual variations in sensory processing with respect to tendencies of behavioral responses to various sensory stimuli such as visual, auditory, touch, taste/smell, movement, and activity level^8^. Previous studies have also reported that sensory profiles help determine individual variations in sensory processing. Although sensory profiling is highly useful in evaluating individual variation in sensory processing, the use of questionnaires for this purpose is associated with difficulty in self–monitoring^9^. Individuals with low intelligence level may not be able to understand the questions while undergoing questionnaire-based sensory profiling; consequently, their sensory profile may not appropriately reflect their sensory processing. Additionally, a previous study showed that a lack of self–monitoring was observed even in individuals with normal intelligence level^10^.

At present, we have not established an objective evaluation method of sensory processing, but magnetic resonance imaging (MRI) has the potential to evaluate sensory processing objectively. MRI is one of the most powerful and flexible imaging tools for evaluating brain tissue. Using task-based MRI, a previous study indicated that sensory stimuli simultaneously activated multiple cortices and suggested that multiple cortices were associated with sensory processing from the perspective of integrating sensory stimuli^11,12^. This relationship indicates that white matter may have an important role in sensory processing because brain white matter tracts connect multiple cortices^13,14^. Brain white matter tracts, which are myelinated axonal fibers, connect multiple different regions such as the primary sensory cortices, higher cortices, deep gray nuclei, and brain stem^15^. Different white matter networks would partially lead to interindividual variability and disorders in sensory processing and resultant behavior responses^16,17^. Numerous studies have examined normal and abnormal connectivity and their role in developmental and acquired disorders such as autism spectrum disorder (ASD)^18^. The development of white matter is susceptible to environmental influences and changes^19^, and previous studies have suggested a relationship between sensory processing and white matter in children with sensory processing disorders^16,17^. Conversely, in adults, the relationship between sensory processing and white matter microstructures remains largely unknown. Moreover, white matter microstructures were shown to differ in each developmental stage and develop differently in each brain region^20^, suggesting that the relationship between sensory processing and white matter microstructures differs in adults.

To evaluate white matter microstructures, we used diffusion tensor imaging (DTI). Recently, using DTI to further investigate white matter anatomy has increased^21^. DTI is based on diffusion-weighted imaging and enables mapping of the diffusion process of water or other molecules. Using appropriate magnetic field gradients, DTI visualizes the white matter tract architecture in the direction of maximum diffusivity aligning with the direction of white matter tract orientation and their myelination, which act as barriers to the random motion of water molecules^18^. Moreover, DTI measures the displacement of water molecules on the micron scale and yields four diffusion parameters: fractional anisotropy (FA), axonal diffusivity (AD), radial diffusivity (RD), and mean diffusivity (MD). These diffusion parameters provide information on white matter tracts such as cell density, axons, and myelin sheath^22^. Here, we investigated the association between the Adolescent/Adult Sensory Profile questionnaire (AASP) and DTI white matter tract architecture. In young adults, the DTI analysis revealed a positive relationship between AASP scores (i.e., sensory sensitivity, sensation avoiding, activity level)/subscores (i.e., sensory sensitivity–activity level and sensation avoiding–touch) and DTI diffusion parameters (i.e., AD and MD) in the cingulum–cingulate gyrus bundle (CCG) and between sensory sensitivity–auditory and MD in the uncinate fasciculus (UNC).

## Results

### Demographics

The present study included 84 typically developed young adults (40 men and 42 women [for intelligence quotient (IQ) scoring]). We could not measure the IQ of two participants. The information on participants, such as age, body mass index (BMI), and IQ, is shown in Table 1.

**Table 1:** Demographic characteristics.

Values		Mean	Standard deviation	Range	Possible score	Cutoff score (age)	
Number (N)		84					
(male, female)		(42, 42)					
Age		24.5	4.7	19–39			
BMI		21.6	2.7	17.0–29.8			
Full scale IQ^a^		114	10	83–135			
**Adolescent/adult sensory profile**							
*Modality-specific subscale*							
Taste/smell		16.7	3.7	8–25	10–50		
Movement		17.1	3.4	9–26	11–55		
Visual		23.1	4.4	11–34	13–65		
Touch		29.0	6.4	15–48	8–40		
Activity level		26.6	4.7	14–37	8–40		
Auditory		22.4	5.1	12–39	10–50		
*Quadrant scores*						(18–34)	(35–64)
Low registration		27.5	6.8	15–48	15–75	23–38	20–34
Sensation seeking		40.5	7.6	23–56	15–75	30–47	29–45
Sensory sensitivity		33.5	7.2	19–51	15–75	25–42	22–39
Sensation avoiding		33.3	7.1	18–47	15–75	25–41	22–39

*BMI* body mass index, *IQ* intelligence quotient.

^a^n = 82.

### Correlations between AASP scores and diffusion parameters of each white matter pathway

**Figure 1﻿:**
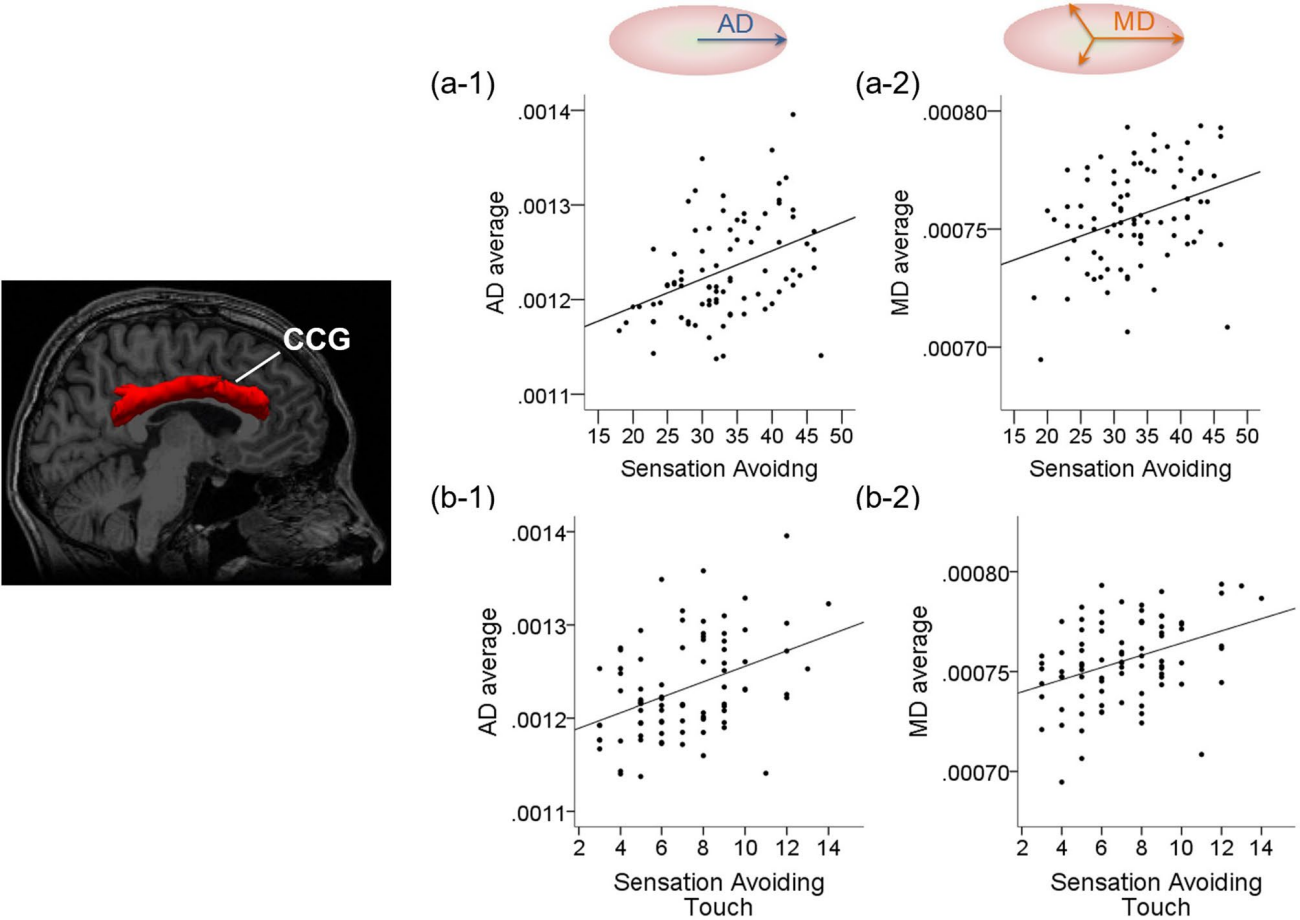
Relationship between right CCG parameters and sensory processing with regard to avoidance of a particular sensation and tactile stimulus. Regarding the right CCG, we observed a positive correlation between diffusion parameters and the AASP score (*P* < *0.001*). (**a**) Correlations between the right CCG and the AASP score (sensation avoiding). (**a-1)** shows the correlation between AD and sensation avoiding, and (**a-2)** shows the correlation between MD and sensation avoiding. (**b**) Correlations between right CCG parameters and the subscore of AASP (sensation avoiding–touch). (**b-1)** shows the correlation between AD and sensation avoiding-touch, and (**b-2)** shows the correlation between MD and sensation avoiding-touch. The right CCG is the red region of interest selected by TRACULA. *CCG* cingulum–cingulate gyrus, *AD* axonal diffusivity, *MD* mean diffusivity, *AASP* Adolescent/Adult Sensory Profile.

**Figure 2:**
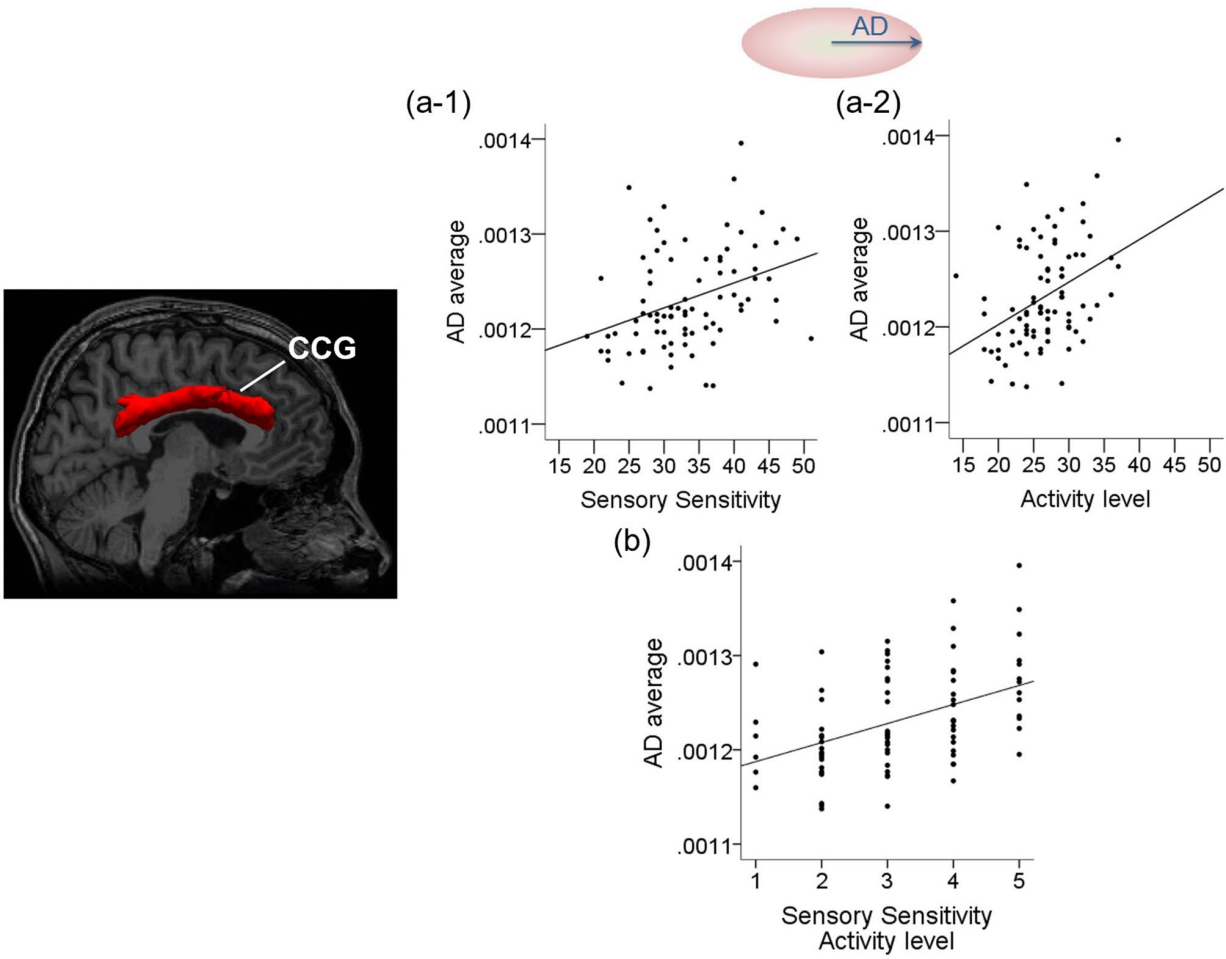
Relationship between the CCG and sensory processing with regard to sensitivity of a particular sensation and activity level-related stimulus. Regarding the right CCG, we observed a positive correlation between diffusion parameters and the AASP score (*P* < *0.001*). (**a**) Correlations between the right CCG and the AASP score (activity level and sensory sensitivity). (**a-1)** shows the correlation between AD and activity level, and (**a-2)** shows the correlation between AD and sensory sensitivity. (**b**) Correlation between right CCG parameters and the subscore of AASP (sensory sensitivity–activity level). Correlation between AD and sensory sensitivity–activity level. The right CCG is the red region of interest selected by TRACULA. *CCG* cingulum-cingulate gyrus, *AD* axonal diffusivity, *MD* mean diffusivity, *AASP* Adolescent/Adult Sensory Profile.

The analysis of AASP scores and diffusion parameters demonstrated positive correlations in the right CCG (*P* < 0.001). The correlations and effect sizes are shown in Table 2, Figs. 1, and 2. In other brain regions, there were no significant correlations between the diffusion parameters and AASP item scores. The correlations corresponding to the right CCG were between: AD and activity level, AD and sensory sensitivity, AD and sensation avoiding, and MD and sensation avoiding. Additionally, we performed a partial correlation analysis, entering age, sex, and BMI as covariates, and found similar results.

**Table 2:** Relationship between diffusion parameters and AASP score.

White matter pathway	Diffusion tensor index	Adolescent/adult sensory profile	Correlation coefficient	Effect size
**Modality**				
Rh_CCG	AD	Activity level	0.390*	0.412
**Quadrant**				
	AD	Sensory sensitivity	0.382*	0.402
	AD	Sensation avoiding	0.421*	0.449
	MD	Sensation avoiding	0.367*	0.385

*Rh_CCG* right cingulum–cingulate gyrus bundle, *AD* axonal diffusivity, *MD* mean diffusivity, *AASP* Adolescent/Adult Sensory Profile.

*P* < 0.001. Effect size was calculated by Fisher's Z_r_.

### Correlations between AASP subscores and diffusion parameters of each white matter pathway

**Figure 3:**
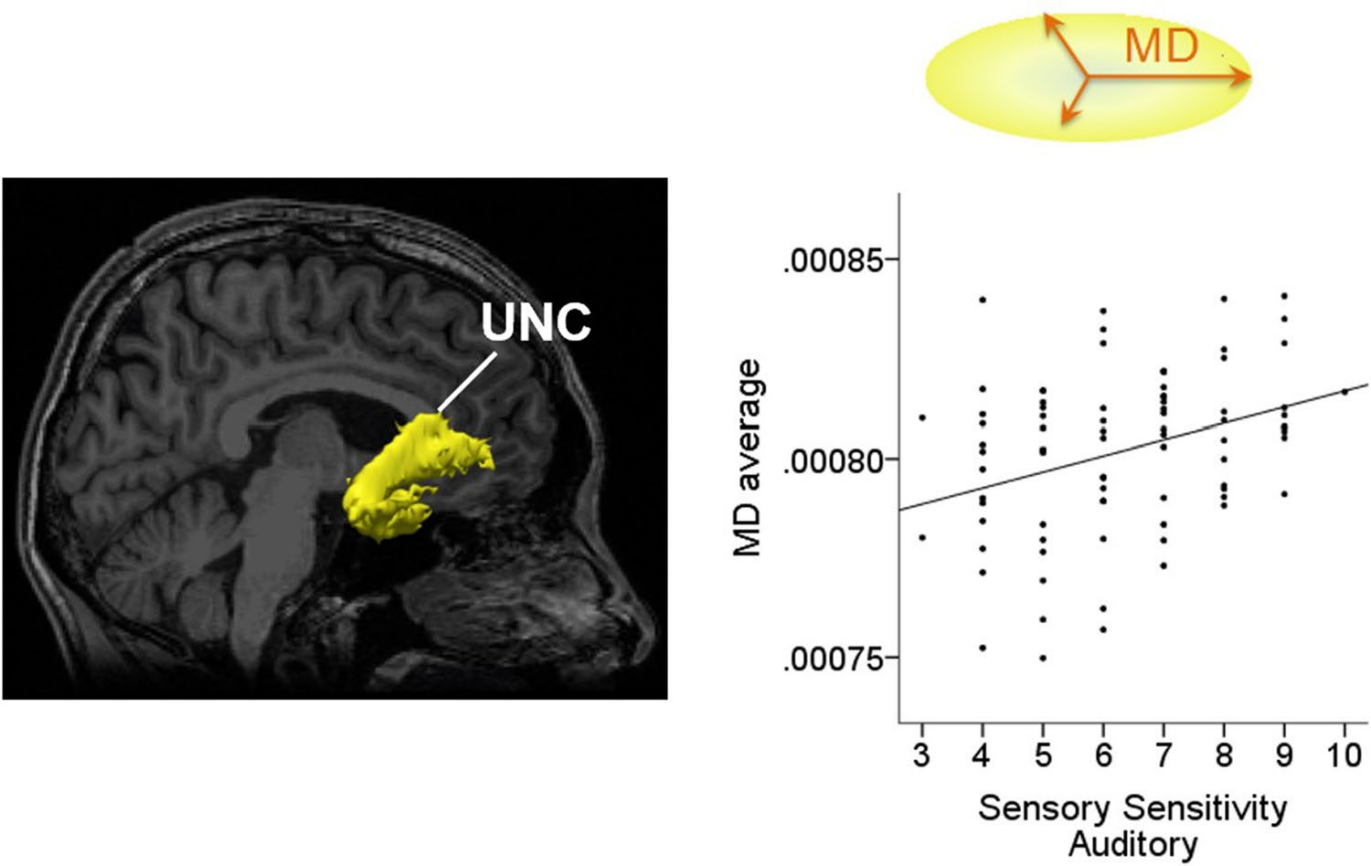
Relationship between the right UNC and sensory processing. In the right UNC, we observed a positive correlation between MD and sensory sensitivity–auditory*.* The right UNC is the yellow region of interest selected by TRACULA. *UNC* uncinate fasciculus, *MD* mean diffusivity, *AASP* Adolescent/Adult Sensory Profile.

Further, the analysis of AASP subscores and diffusion parameters demonstrated positive correlations in the right CCG and right UNC (*P* < *0.001*). The correlations corresponding to the right CCG were between: AD and sensory sensitivity–activity level, AD and sensation avoiding–touch, and MD and sensation avoiding–touch. Additionally, the correlation corresponding to the right UNC was between MD and sensory sensitivity–auditory. Correlations and effect sizes are shown in Table 3 and Figs. 1, 2 and 3. In other brain regions, there were no significant correlations between diffusion parameters and AASP subscores. Additionally, we performed a partial correlation analysis, entering age, sex, and BMI as covariates, and found similar results.

**Table 3:** Relationship between diffusion parameters and subscores of AASP.

White matter pathway	Diffusion tensor index	Subscore of AASP	Correlation coefficient	Effect size
Rh_CCG	AD	Sensory sensitivity-activity level	0.442*	0.475
	AD	Sensation avoiding-touch	0.425*	0.454
	MD	Sensation avoiding-touch	0.385*	0.406
Rh_UNC	MD	Sensory sensitivity-auditory	0.365*	0.382

*Rh_CCG* right cingulum-cingulate gyrus bundle, *Rh_UNC* right uncinate fasciculus, *AD* axonal diffusivity, *MD* mean diffusivity, *AASP* Adolescent/adult sensory profile.

*P < 0.001. Effect size was calculated by Fisher's Z_r_.

## Discussion

The present study revealed a positive correlation between diffusion parameters of the right CCG (i.e., AD, MD) and AASP scores (i.e., sensation avoiding, sensory sensitivity, activity level). Additionally, we found positive correlations between the right CCG (i.e., AD, MD) and subscores of AASP (i.e., sensation avoiding–touch, sensory sensitivity–activity level) and between MD of the right UNC and sensory sensitivity–auditory. We evaluated the effect sizes of these correlations as moderate or strong. The present study suggested that variability in white matter microstructures in the CCG and UNC may indicate low neurological thresholds for sensory stimuli in young adults.

In studies using DTI, the interpretation of diffusion parameters is important. The pathophysiology of each diffusion parameter has been revealed by previous studies^23–25^. FA is sensitive to changes in white matter microstructure^23^. Previous studies involving typical developed participants have demonstrated FA increases with growth, suggesting that myelin is the most influential factor of white matter microstructure anisotropy^24–26^. The present study showed no significant correlation between FA and sensory processing, suggesting there were no differences in myelin with respect to sensory processing. Additionally, RD demonstrated no significant correlation with sensory processing in the present study. This reinforces that myelin has no relationship with sensory processing, because RD is an eigenvector perpendicular to axons and is sensitive to changes in myelination^25,27^. We also found a correlation between AD in the CCG and sensory processing. AD is an eigenvector of diffusion parameters in the axonal direction^25,27^. Previous studies have suggested that AD is sensitive to axonal disassembly and that an increase in AD is caused by a decrease in the tortuosity of white matter tracts in the axonal direction, which involves changes in maturation, from tortuous to straight^25,26^. Thus, AD was considered to indicate tract-specific or timing-specific changes in axonal morphology^25^. The CCG presented a stronger decrease in axon tortuosity than other white matter regions in the course of brain development^28^. The present CCG results suggest that axonal changes, such as those in axon tortuosity with brain development, have an important role in the presentation of sensitivity to sensory stimuli. Furthermore, the present study showed that MD of the CCG and the UNC were correlated with sensory processing. MD is the mean of diffusivity in the axonal and perpendicular direction to axons and is affected by the size and integrity of cells^23^. In conjunction with the positive correlation between AD of the CCG and sensory processing, we suggest the integrity of the CCG in the axonal direction is related to sensory processing. In the UNC, we found a positive correlation between MD and sensory sensitivity–auditory. The UNC is the last region to mature during development^28^ and MD decreases with growth^23^. Thus, high MD is assumed to represent immaturity of nerve fibers, and we suggest that immaturity of the UNC is related to sensory sensitivity to auditory stimuli.

The present study showed a positive correlation between diffusion parameters of the right CCG and AASP scores/subscores. Using effect sizes based on previous studies^29,30^, in the CCG, almost all correlations were moderate; however, the correlation was strong for sensation avoiding–touch and sensory sensitivity–activity level. A previous study indicated that the CCG connects various areas: the cingulate cortex, orbitofrontal cortex, splenium of the corpus callosum, parahippocampal gyrus, superior frontal gyrus, precuneus, superior parietal lobule, superior occipital lobule, and supplementary motor area^31^. The CCG is considered a core part of the limbic system and to be involved in the processing of emotion, pain, reward, motion, conflict, error detection, and memory, which mainly reflect the involvement of the cingulate cortex^32,33^. Another previous study indicated that the anterior cingulate cortex and insula were activated by sensory stimuli regardless of sensory modality, such as somatosensory, auditory, or visual; new sensory stimuli increased the activation in these areas^34^. Among the brain areas involved in the CCG, research has suggested that the anterior cingulate cortex and insula formed a network for the detection of changes in sensory stimuli ^34,35^. The present results showed that diffusion parameters of the CCG were correlated with sensation avoidance and sensitivity, which are inverse behavioral responses but have low neurological thresholds for sensory stimuli according to Dunn’s theory. In conjunction with the interpretation of diffusion parameters, our results suggest that the large axonal variability in the CCG were involved in the low neurological threshold for the sensory stimuli without behavioral responses to sensory stimuli.

Moreover, we observed that CCG parameters were related to the activity level of the AASP score and sensory sensitivity–activity level of the AASP subscore. Activity level-related items measure disposition toward involvement in daily activities^36^ and sensory sensitivity–activity level indicates excessive response to environmental sensory stimuli in daily life and behavioral features of distractedness. The environmental sensory stimuli represented by activity level consist of multisensory stimuli such as visual and auditory stimuli in daily life. A previous study suggested that multisensory processing was related to the brain area centered at the CCG^37^. Additional previous studies have suggested that the CCG is related to attention to the surroundings in daily life and sustained attention^38–40^. Considering this relationship between the CCG and multisensory processing or attention and diffusion parameters, our results suggest that large axonal change in the CCG are related to a low neurological threshold to environmental stimuli in daily life and control of attention to sensory stimuli.

Additionally, we demonstrated that the CCG had a relationship with sensation avoiding–touch. Touch processing-related items in the AASP measure responses to tactile stimuli on the skin. The relationship between the CCG and tactile stimuli was previously observed in gentle touch-induced activities in the somatosensory, motor area, and cingulate regions^41^ and non-self/self-produced tactile stimulus-induced/reduced activities in the cingulate gyrus^42^. Thus, in conjunction with previous findings, we suggest that CCG parameters are related to a low neurological threshold for processing of physical touch.

In the present study, a right UNC parameter demonstrated a moderate correlation with sensory sensitivity–auditory. The UNC not only connects the orbitofrontal cortex and temporal lobe anatomically, but also is considered the key pathway associated with episodic memory, language, and social emotional processing^43,44^. A recent meta-analysis of 25 DTI-based studies indicated that individuals with ASD showed decreased FA in the UNC, thus supporting the theory of specific underconnectivity in patients with ASD who show difficulty in social auditory information and language processing^45^. Furthermore, a previous mouse model showed that the UNC was anatomically connected to the primary auditory cortex and activated by sound stimuli and stimuli to the orbitofrontal cortex. Thus, the UNC and auditory processing are closely intertwined. Auditory processing items measure response to stimuli that are heard by an individual. Sensory sensitivity–auditory is associated with high sensitivity to auditory stimuli, leading to the presentation of excessive behavioral responses and disturbing behavior^36^. Our present results suggest that the UNC is a core white matter connection between the orbitofrontal cortex and primary auditory cortex and of the interaction related to sensitivity to auditory stimuli and behavioral response.

The present study has several limitations. First, the effect of age should be considered. Previous studies have shown that the maturity of white matter differs in each brain area and that the CCG and UNC develop later than other white matter tracts^33,46^. The peak of CCG FA was observed at 42 years of age; therefore, the CCG was considered one of the white matter tracts that mature at a later stage^46^. Additionally, the UNC is one of the last developing tracts, and it continues maturing into the third decade of life^47^. The maturational peak of the UNC is extended throughout adolescence and young adulthood and peaks beyond the age of 30 years^46^. Considering that the present study targeted young adulthood, the present results suggest that the degree of maturity of the CCG and UNC may affect sensory processing in young adulthood. In the present study, although we considered age as a control variable, it should be noted that the age range was 19–39 years with an average of 24.5 years. Our results should not be applied to other developmental stages. Notably, previous studies where the ages of the subjects were 8–12 or 8–11 years showed that there was a relationship between sensory processing and posterior white matter related to motor and sensory areas, which contrasts our lack of results^16,17^. Considering that the maturation speed of white matter is different in each brain area, this finding does not contradict the hypothesis that brain areas related to sensory processing differ at each development stage.

Additionally, there was no psychometric support for the use of the AASP subscores. The AASP is designed to evaluate sensory processing in four quadrants and six modalities. Thus, we should use caution when interpreting the AASP subscores. Certainly, the AASP is a useful tool to examine sensory processing, but the AASP score cannot explain, in detail, the effect of a modality on a quadrant. Because using the AASP as originally designed is insufficient to research sensory processing in detail, we evaluated sensory processing in using the AASP subscores.

In future studies, because our results can only be generalized to young, typically developed adults, we should conduct research regarding disorders that affect sensory processing, such as ASD. ASD is a complex neurodevelopmental condition with variable deficits in social behavior and language, restrictive interests, repetitive behaviors, and sensory processing^48,49^. It has been reported that 94% of individual with ASD show sensory symptoms, which are suggested to have a central role in other symptoms, such as social–communication deficits and repetitive behaviors^48,49^. Considering that differences in white matter microstructure have been reported in ASD^50^, our results in young, typically developed adults may not be applicable to this population. The relationship between sensory processing and white matter microstructures using AASP and DTI in a sample of individuals with ASD needs to be examined. As differences between ASD and typical development have been indicated in the CCG and UNC^50^, we hypothesize that ASD would demonstrate different relationships between sensory processing and white matter microstructures from those reported here.

The present study revealed the relationship between sensory processing and CCG and UNC white matter parameters. We suggest that in young adults, the axonal tortuosity of the CCG represents a low neurological threshold regardless of behavioral responses to sensory stimuli and that the cell integrity of the UNC is related to sensory sensitivity to auditory stimuli. While we need more studies involving participants in other development stages to interpret the diffusion tensor index, our current study has provided new information on the neurological basis of sensory processing.

## Methods

### Participants

The present study included 84 typically developed individuals (42 men and 42 women); they were right handed, as assessed with the Edinburgh Handedness Inventory^51^. Participants who were using psychiatric medications at the time of evaluation or had a history of brain injury, major medical conditions, or alcohol or drug dependence were excluded. Full-scale IQ scores were measured using the Wechsler Adult Intelligence Scale-III. Our study was performed in accordance with the Health Insurance Portability and Accountability Act guidelines, was approved by the Research Ethics Committee of University of Fukui, and all participants provided written informed consent. Typically developed participants were interviewed by trained psychiatrists (H.K., I.O) to screen for the presence of neuropsychiatric disorders using the Structured Clinical Interview for the Diagnostic and Statistical Manual of Mental Disorders, fourth edition—Axis I Disorders^52^.

#### AASP

We used the Japanese version of the AASP for the evaluation of sensory processing^53^. The AASP was standardized in Japanese and showed comparable psychometric properties with the original instrument^53,54^. The AASP is a standardized self-report questionnaire that includes 60 items in total and employs a five-point Likert scale. The AASP consists of sensory processing modalities and quadrants and includes six modalities, namely visual, auditory, touch, smell/taste, movement, and activity level, composed of 10, 11, 13, 8, 8, and 10 items, respectively. The sum of scores of modality-specific items was determined; the higher the total score of a modality, the greater the corresponding atypical tendencies of sensory processing. Moreover, the AASP includes four quadrant scores, each consisting of 15 items: low registration, sensation seeking, sensory sensitivity, and sensation avoiding. The quadrant states are based on Dunn’s sensory processing model^36^, which classifies the tendency of responses to sensory stimuli in terms of a neurological threshold and behavioral responses. Low registration refers to the tendency of showing a high neurological threshold and passive behavioral responses, associated with sensory bluntness where even strong sensory stimuli may go unnoticed. This tendency is associated with a delay in response to sensory stimuli and often with passive behavioral response. Sensation seeking refers to the tendency of showing a high neurological threshold and active behavioral responses. Sensation seeking requires specific sensory stimuli to satisfy a high neurological threshold and stabilize an easily bored state without specific sensory stimuli. Sensory sensitivity refers to the tendency of showing a low neurological threshold, receiving strong stimuli, and feeling pain even after exposure to mild sensory stimuli because of passive behavioral responses. Sensation avoiding refers to the tendency of showing low neurological thresholds and avoiding unpleasant sensory stimuli because of active behavioral responses. In a previous standardization study in Japanese version of the AASP, typical sensory processing was demonstrated in 16–84% of the research sample^53^. The cutoff scores for participants aged 18–34 years were 23–38 in low registration, 30–47 in sensation seeking, 25–42 in sensory sensitivity, and 25–41 in sensation avoiding^53^. The cutoff scores for participants aged 35–64 years old were 20–34 in low registration, 29–45 in sensation seeking, 22–39 in sensory sensitivity, and 22–39 in sensation avoiding^53^. We found that the mean for all quadrant scores were within the cutoff score. Although four participants were beyond the cutoff scores, we included all participants in our statistical analysis because all participants were evaluated to be typically developed by trained psychiatrists (H.K., I.O). Information on each modality-specific and quadrant score (mean, standard deviation, range, and possible score) of participants is shown in Table 1.

To study sensory processing in further detail, we subdivided modality-specific items according to quadrant. For example, for the item “I am distracted if there is a lot of noise around”, the corresponding quadrant modality is sensory sensitivity–auditory. The sum of quadrant-specific modality scores was considered the subscore. There were 24 subscores, which had 1–4 items each. The sum of the subscores was determined; the higher the total subscore, the greater the quadrant tendencies of the modality-specific sensory processing.

### MRI acquisition

MR images were acquired using a 3-Tesla positron emission tomography (PET)/MR scanner (SIGNA PET/MR; General Electric Medical Systems, Milwaukee, WI, USA) with an 8-channel head coil. High-resolution T1-weighted anatomical MRI and DTI were acquired using the scan parameters of our previous study^55^. The detailed scan parameters were: T1-weighted anatomical MRI (repetition time = 6.38 ms, echo time (TE) = 1.99 ms, flip angle = 11°, field of view = 256 mm, number of slices, 172, voxel dimension = 1.0 × 1.0 × 1.0 mm^3^); DTI (single–shot echo–planar imaging, acquisition matrix = 128 × 128, TE = minimum, repetition time = 9327 ms, field of view = 240 mm, pixel size = 1.9 mm × 1.9 mm^2^, number of axial slices = 45, slice thickness/gap = 3.0 mm/0 mm, 30 distributed isotropic orientations, b–values of 1000 s/mm^2^ and 0).

### DTI analysis

We used TRActs Constrained by UnderLying Anatomy (TRACULA^56^) for the analysis of diffusion–weighted images. TRACULA is an automated global probabilistic tractography tool in FreeSurfer (version 6.0; http://surfer.nmr.mgh.harvard.edu/). First, we reconstructed each participant’s T1-weighted anatomical image and obtained cortical and subcortical regions using FreeSurfer^57^. Subsequently, we used TRACULA to reconstruct volumetric distributions of 18 major white matter pathways in the participants’ diffusion-weighted data. The automated tractography method relies on prior anatomical information based on a set of training subjects, which was obtained with manual labeling. The 18 reconstructed white matter pathways were as follows: the bilateral corticospinal tract, bilateral inferior longitudinal fasciculus, bilateral UNC, bilateral anterior thalamic radiations, bilateral CCG, bilateral cingulum–angular bundle, bilateral superior longitudinal fasciculus–parietal terminations, bilateral superior longitudinal fasciculus–temporal terminations, corpus callosum–forceps major, and corpus callosum–forceps minor. Finally, we extracted four diffusion parameters (FA, MD, RD, and AD) from each white matter pathway using the “ball-and-stick” model of diffusion. The mean values of diffusion measures were calculated by thresholding each pathway distribution at 20% of its maximum value for each participant. To quantify head motion in each participant’s diffusion-weighted data, we calculated four measures of head motion: translation (mm), rotation (degree), average signal intensity dropout score, and portion of slices with greater than the computed dropout score (%) and removed eddy current-induced image distortions^58^.

### Statistical analysis

We performed partial correlation analysis using SPSS (version 20; IBM Corp., Armonk, NY, USA) to evaluate the strength of the relationship between the AASP score (six modality-specific scores, four quadrant scores) and diffusion measures (FA, MD, AD, and RD) of each white matter pathway, by considering age, BMI, and sex-specific differences as control variables. Additionally, to examine the AASP in further detail, we performed a partial correlation analysis to evaluate the relationship between the AASP subscores (24 items; low registration–visual, low registration–auditory, etc.) and diffusion parameters of each white matter pathway using age, BMI, and sex differences as control variables. In all partial correlation analyses, *P* < 0.001 was considered statistically significant. Additionally, we calculated Fisher’s Z_*r*_ of the statistically significant correlations as the effect size^59^ and evaluated effect sizes based on previous studies^29,30^.

## Data Availability

The datasets generated and/or analyzed during the current study are available from the corresponding author on reasonable request.

## References

[1] Kilroy E, Aziz-Zadeh L, Cermak S (2019). Ayres theories of autism and sensory integration revisited: What contemporary neuroscience has to say. Brain Sci..

[2] Thye MD, Bednarz HM, Herringshaw AJ, Sartin EB, Kana RK (2018). The impact of atypical sensory processing on social impairments in autism spectrum disorder. Dev. Cogn. Neurosci..

[3] Miller LJ, Schoen SA, James K, Schaaf RC (2007). Lessons learned: A pilot study on occupational therapy effectiveness for children with sensory modulation disorder. Am. J. Occup. Ther..

[4] Ben-asson A (2008). Sensory clusters of toddlers with autism spectrum disorders: Differences in affective symptoms. J. Child Psychol. Psychiatry Allied Discip..

[5] Jasmin E (2009). Sensori-motor and daily living skills of preschool children with autism spectrum disorders. J. Autism Dev. Disord..

[6] Dunn W (1997). The impact of sensory processing abilities on the daily lives of young children and their families: A conceptual model. Infants Young Child..

[7] Metz AE (2019). Dunn’s model of sensory processing: An investigation of the axes of the four-quadrant model in healthy adults. Brain Sci..

[8] Brown C, Tollefson N, Dunn W, Cromwell R, Filion D (2001). The adult sensory profile: measuring patterns of sensory processing. Am. J. Occup. Ther..

[9] Yoshimura S (2017). Gray matter volumes of early sensory regions are associated with individual differences in sensory processing. Hum. Brain Mapp..

[10] Huang AX (2017). Understanding the self in individuals with autism spectrum disorders (ASD): A review of literature. Front. Psychol..

[11] Krauss P, Tziridis K, Schilling A, Schulze H (2018). Cross-modal stochastic resonance as a universal principle to enhance sensory processing. Front. Neurosci..

[12] Green SA (2015). Neurobiology of sensory overresponsivity in youth with autism spectrum disorders. JAMA Psychiatr..

[13] Wu X (2017). Functional connectivity and activity of white matter in somatosensory pathways under tactile stimulations. Neuroimage.

[14] Peer M, Nitzan M, Bick AS, Levin N, Arzy S (2017). Evidence for functional networks within the human brain’s white matter. J. Neurosci..

[15] Mori S, Oishi K, Faria AV (2009). White matter atlases based on diffusion tensor imaging. Curr. Opin. Neurol..

[16] Owen JP (2013). Abnormal white matter microstructure in children with sensory processing disorders. NeuroImage Clin..

[17] Chang YS (2016). White matter microstructure is associated with auditory and tactile processing in children with and without sensory processing disorder. Front. Neuroanat..

[18] Wycoco V, Shroff M, Sudhakar S, Lee W (2013). White matter anatomy: What the radiologist needs to know. Neuroimaging Clin. N. Am..

[19] Lebel C, Deoni S (2018). The development of brain white matter microstructure. Neuroimage.

[20] Tamnes CK (2010). Brain maturation in adolescence and young adulthood: Regional age-related changes in cortical thickness and white matter volume and microstructure. Cereb. Cortex.

[21] Winklewski PJ (2018). Understanding the physiopathology behind axial and radial diffusivity changes—What do we know?. Front Neurol..

[22] Assaf Y, Pasternak O (2008). Diffusion tensor imaging (DTI)-based white matter mapping in brain research: A review. J Mol Neurosci..

[23] Tae WS, Ham BJ, Pyun SB, Kang SH, Kim BJ (2018). Current clinical applications of diffusion-tensor imaging in neurological disorders. J. Clin. Neurol..

[24] Lebel C, Beaulieu C (2011). Longitudinal development of human brain wiring continues from childhood into adulthood. J. Neurosci..

[25] Lebel C, Treit S, Beaulieu C (2019). A review of diffusion MRI of typical white matter development from early childhood to young adulthood. NMR Biomed..

[26] Beaulieu C (2002). The basis of anisotropic water diffusion in the nervous system - A technical review. NMR Biomed..

[27] Aung WY, Mar S, Benzinger TL (2013). Diffusion tensor MRI as a biomarker in axonal and myelin damage. Imaging Med..

[28] Simmonds DJ, Hallquist MN, Asato M, Luna B (2014). Developmental stages and sex differences of white matter and behavioral development through adolescence: A longitudinal diffusion tensor imaging (DTI) study. Neuroimage.

[29] Nakagawa S, Cuthill IC (2007). Effect size, confidence interval and statistical significance: A practical guide for biologists. Biol. Rev..

[30] Cohen, J. *Statistical Power Analysis for the Behavioral Sciences* (2nd edn). Hillsdale, NJ: Lawrence Erlbaum Associates (2013).

[31] Wu Y, Sun D, Wang Y, Wang Y, Ou S (2016). Segmentation of the cingulum bundle in the human brain: A new perspective based on DSI tractography and fiber dissection study. Front. Neuroanat..

[32] Bubb EJ, Kinnavane L, Aggleton JP (2017). Hippocampal - diencephalic - cingulate networks for memory and emotion: an anatomical guide. Brain Neurosci. Adv..

[33] Bubb EJ, Metzler-Baddeley C, Aggleton JP (2018). The cingulum bundle: Anatomy, function, and dysfunction. Neurosci. Biobehav. Rev..

[34] Downar J, Crawley AP, Mikulis DJ, Davis KD (2000). A multimodal cortical network for the detection of changes in the sensory environment. Nat. Neurosci..

[35] Mouraux A, Diukova A, Lee MC, Wise RG, Iannetti GD (2011). A multisensory investigation of the functional significance of the “pain matrix”. Neuroimage.

[36] Brown, C. & Dunn, W. *Adolescent-Adult Sensory Profile: User's Manual*. San Antonio, TX: Psychological Corporation (2002).

[37] Huang S (2015). Multisensory competition is modulated by sensory pathway interactions with fronto-sensorimotor and default-mode network regions. J. Neurosci..

[38] Leech R, Sharp DJ (2014). The role of the posterior cingulate cortex in cognition and disease. Brain.

[39] Van Den Heuvel, M., Mandl, R., Luigjes, J. & Hulshoff Pol, H. Microstructural organization of the cingulum tract and the level of default mode functional connectivity. *J. Neurosci.* **28**, 10844–10851 (2008). 10.1523/JNEUROSCI.2964-08.2008 PMC6671361 18945892

[40] Bonnelle V (2011). Default mode network connectivity predicts sustained attention deficits after traumatic brain injury. J. Neurosci..

[41] Eriksson Hagberg, E. *et al*. Spatio-temporal profile of brain activity during gentle touch investigated with magnetoencephalography. *Neuroimage* **201**, 116024 (2019). 10.1016/j.neuroimage.2019.116024 31323258

[42] Boehme R, Hauser S, Gerling GJ, Heilig M, Olausson H (2019). Distinction of self-produced touch and social touch at cortical and spinal cord levels. Proc. Natl. Acad. Sci. U. S. A..

[43] Bhatia K, Henderson L, Yim M, Hsu E, Dhaliwal R (2017). Diffusion tensor imaging investigation of uncinate fasciculus anatomy in healthy controls: Description of a subgenual stem. Neuropsychobiology.

[44] Von Der Heide RJ, Skipper LM, Klobusicky E, Olson IR (2013). Dissecting the uncinate fasciculus: Disorders, controversies and a hypothesis. Brain.

[45] Chang, Y. S. *et al*. Autism and sensory processing disorders: Shared white matter disruption in sensory pathways but divergent connectivity in social-emotional pathways. *PLoS One* **9**, e103038 (2014). 10.1371/journal.pone.0103038 PMC4116166 25075609

[46] Lebel C (2012). Diffusion tensor imaging of white matter tract evolution over the lifespan. Neuroimage.

[47] Olson IR, Von Der Heide RJ, Alm KH, Vyas G (2015). Development of the uncinate fasciculus: Implications for theory and developmental disorders. Dev. Cogn. Neurosci..

[48] Robertson CE, Baron-Cohen S (2017). Sensory perception in autism. Nat. Rev. Neurosci..

[49] Geschwind DH (2009). Advances in autism. Annu. Rev. Med..

[50] Travers BG (2012). Diffusion tensor imaging in autism spectrum disorder: A review. Autism Res..

[51] Oldfield RC (1971). The assessment and analysis of handedness: The Edinburgh inventory. Neuropsychologia.

[52] First, M.B., Spitzer, R. L., Gibbon, M., & Williams Janet, B. W. *Structured Clinical Interview for DSM-IV Axis I Disorders*. Washington, DC: American Psychiatric Press (1997).

[53] Brown, C. & Dunn, W. *The Japanese Version of Adolescent/Adult Sensory Profile: User’s Manual [in Japanese]*. Tokyo, Japan: Nihonbunnkakagakusya (2015).

[54] Ito H (2013). Standardization of the Japanese version of the sensory profile: Reliability and norms based on a community sample. Seishinigaku.

[55] Jung M (2019). Sex differences in white matter pathways related to language ability. Front. Neurosci..

[56] Yendiki A (2011). Automated probabilistic reconstruction of white-matter pathways in health and disease using an atlas of the underlying anatomy. Front. Neuroinform..

[57] Dale AM, Fischl B, Sereno MI (1999). Cortical surface-based analysis I. Segmentation and surface reconstruction. Neuroimage.

[58] Yendiki A, Koldewyn K, Kakunoori S, Kanwisher N, Fischl B (2014). Spurious group differences due to head motion in a diffusion MRI study. Neuroimage.

[59] Lipsey M. W. & Wilson D. B. Practical Meta-Analysis. Applied Social Research Methods Series, Vol. 49, SAGE Publications, Thousand Oaks, CA (2000).

